# Editorial: Helminth biodiversity

**DOI:** 10.3389/fvets.2022.1126460

**Published:** 2023-01-19

**Authors:** Raquel de Oliveira Simões, María del Rosario Robles

**Affiliations:** ^1^Departamento de Parasitologia Animal, Universidade Federal Rural Do Rio de Janeiro, Seropédica, Brazil; ^2^Centro Científico Tecnológico La Plata (CONICET-UNLP), Facultad de Ciencias Naturales y Museo, Centro de Estudios Parasitológicos y de Vectores, Buenos Aires, Argentina

**Keywords:** parasitology, taxonomy, ecology, phylogeny, biology

Biodiversity conservation has been a global concern, mainly due to the growing disturbance in the structure and function of ecosystems (1). Consequently, this results in the loss of native diversity, including parasites from different vertebrate hosts (2). In view of this, the knowledge of helminth biodiversity is extremely important, since parasites can also bring relevant information about environmental stress, structure, and function in the food chain and biodiversity (3–5).

The importance of recording biodiversity is compromised by the so-called taxonomy crisis. The purely taxonomic aims, which involve describing species, are conditioned by other aspects of interest, particularly economic-productive and/or sanitary-epidemiological. This situation is reflected in reduced inventories and concentrated representation of certain taxonomic groups. Other situations that are observed are the concentration of specialists in charismatic taxonomic groups, or in those that “inherit” from their trainers, leaving many groups unexplored. There are also the problems of generational replacement since there is little interest from the new generations of biologists in the discipline [e.g., (6, 7)].

In this context, within the framework of the parasitological discipline, the fact that parasitic organisms are a group that is not precisely characterized by concentrating efforts for their conservation is added to this crisis. In contrast, the parasites are usually approached with the objectives of partial eradication, in the case of those of medical-veterinary importance, while the rest, which is an integral part of the ecosystems, represent a very low portion of the goals of interest (8). In addition, it is even more worrying that the taxonomic aspects of different helminth groups are often underestimated and do not have a place in high-impact journals.

The aim of this Research Topic is to broaden and deepen the knowledge about helminths in wildlife or domestic hosts around the world focusing on the contribution to the taxonomic groups, ecology, phylogenetic, and biology. Thus, a multidisciplinary approach is given and can be used as a guide for scientific information. In this special collection, there are seven papers covering the following aspects.

(1) A new species of *Pterygodermatites* (Nematoda: Spirurida) is described as parasitizing the gastrointestinal tract of the didelphid marsupial *Marmosa constantiae* (Andrade-Silva et al.). This marsupial was collected in the transitional phytogeography formed by ecotones from Cerrado/Amazon biomes in the north of the state of Mato Grosso, Brazil. These biomes are characterized by considerable biodiversity of mammals [(9) and little known or characterized parasites in the *M. constantiae*].
(2) The use of integrative taxonomy is a useful tool for the diagnosis of species morphologically similar, mainly in the larval stage. Santoro et al., using scanning electron microscopy and molecular analysis, characterized the larvae of the cestode belonging to the family Gymnorhynchidae. These larvae infect the edible musculature of the Atlantic pomfret in the Mediterranean. The integrative taxonomy allowed for accurate discrimination between larvae of *Molicola uncinatus* and *Gymnorhynchus gigas* in co-infection.
(3) A review of cestodes that parasites Neotropical hystricomorphic rodent was done by Jones. This update aimed to identify the cestodes found in the hunted rodent. Metacestodes of *Echinococcus vogeli* and *Echinococcus oligarthrus* were commonly found in the liver, muscle, and subcutaneous tissue of lappe (*Cuniculus paca*) and agouti (*Dasyprocta leporine*). In contrast, these cestode larvae were not recorded in the tissues of the capybara (*Hydrochoerus hydrochaeris*). This demonstrates the distinct feeding habits among capybara, agouti, and lappe and shows the importance of these animals as intermediate hosts of these zoonotic cestodes.
(4) Synanthropic rodents such as *Mus musculus, Rattus rattus*, and *Rattus norvegicus* affect the ecology of the native communities (10) as well as the epidemiology of some diseases, once infected with parasites (11). Describing the pattern of gastrointestinal helminth infection from these rodents helps to understand the dispersion of helminths. In this work, Grandón-Ojeda examined the helminths from the stomach and intestines of introduced rats in Chile and characterized the association between the abundance of parasites and biotic and abiotic factors.
(5) Many species of mollusks harbor nematodes of medically important and act as paratenic or intermediate hosts (12–14). Thiengo et al. evaluated and summarized the parasitological analyses done by their reference laboratory and reported the presence of nematodes with human and veterinary importance found in terrestrial snails in Brazil. This study is important to the knowledge of parasite transmission and the information produced can be used as a subsidy for control measures and parasite disease transmission, mainly where invasive mollusks are present.
(6) A case report of a female dog infected with aggressive *Dirofilaria immitis* infection is given (Taweethavonsawat et al.). This article describes the clinical signs and procedure to remove the nematode from the heart and erratic places. The histopathology, molecular biology, and technique of necropsy were also reported in this case.
(7) The last article presented in this collection is a study that evaluated the usefulness of matrix-assisted laser desorption/ionization time-of-flight mass spectrometry (MALDI-TOF MS) as an effective diagnostic tool for the specific identification of *Trichuris* (Rivero et al.). The initial study confirmed the efficiency of MALDI-TOF MS as a fast and secure method for the correct identification of *Trichuris* species. Additionally, a MALDI-TOF MS profile of *T. suis* proteome was carried out to develop the first internal database of spectra for the diagnosis of trichuriasis and other *Trichuris* spp.

In summary, the studies and review above mentioned contribute to the knowledge of the taxonomy, biology, ecology, and diagnostic of helminth biodiversity around the world. These reports demonstrate the importance and necessity of studies in several aspects related to helminthology. After reading these articles, the reader will have an overview of helminth biodiversity and how much is not known and still needs to be done.

## Author contributions

MR wrote the introduction and revised the text. RS wrote the central part with comments on the cited articles, conclusions, and references. Both authors contributed to the article and approved the submitted version.

## References

[1] Campos RC Hernández MI. Changes in the dynamics of functional groups in communities of dung beetles in Atlantic forest fragments adjacent to transgenic maize crops. Ecol Indic. (2015) 49:216–27. 10.1016/j.ecolind.2014.09.043

[2] Lafferty KD. Biodiversity loss decreases parasite diversity: theory and patterns. Philos Trans R Soc Lond B Biol Sci. (2012) 367:2814–27. 10.1098/rstb.2012.0110 22966137 PMC3427564

[3] Poulin R. Phylogeny, ecology and the richness of parasite communities in vertebrates. Ecol Monogr. (1995) 65:283–302. 10.2307/2937061

[4] Lafferty KD Allesina S Arim M Briggs CJ Leo G Dobson AP. Parasites in food webs: the ultimate missing links. Ecol Lett. (2008) 11:533–46. 10.1111/j.1461-0248.2008.01174.x 18462196 PMC2408649

[5] Marcogliese DJ. Parasites: Small players with crucial roles in the ecological theater. Ecohealth. (2004) 1:151–64. 10.1007/s10393-004-0028-3

[6] Hołyński RB. Taxonomy crisis, biodiversity disaster–and sabotaging regulations. MunisEnt Zool. (2008) 3:1–6.

[7] Mayo SJ Allkin R Bake W Blagoderov V Brake I Clark B.. Alpha e-taxonomy: responses from the systematics community to the biodiversity crisis. Kew Bulletin. (2003) 63:1–16. 10.1007/s12225-008-9014-1

[8] Poulin R. Parasites and the neutral theory of biodiversity. Ecography. (2004) 27:3–129. 10.1111/j.0906-7590.2004.03695.x

[9] Lacher TE Alho CJR. Terrestrial small mammal richness and habitat associations in an Amazon Forest–Cerrado contact zone. Biotropica. (2001) 33:171–81. 10.1111/j.1744-7429.2001.tb00166.x

[10] Banks PB Hughes NK. A review of the evidence for potential impacts of black rats (Rattus rattus) on wildlife and humans in Australia. Wildlife Res. (2012) 39:78–88. 10.1071/WR11086

[11] Morand S Bordes F Chen H-W Claude J Cosson J-F Galan M. Global parasite and *Rattus* rodent invasions: the consequences for rodent-borne diseases. Integr Zool. (2015) 10:409–23. 10.1111/1749-4877.12143 26037785

[12] Kim JR Hayes KA Yeung NW Cowie RH. Diverse of gastropod hosts of *Angiostrongylus cantonensis*, the rat lungworm, globally and with a focus on the Hawaiian Islands. PLoS ONE. (2014) 9:e94969. 10.1371/journal.pone.0094969 24788772 PMC4008484

[13] Morassutti AL Thiengo SC Fernandez M Sawanyawisuth K Graeff-Teixeira C. Eosinophilic meningitis caused by *Angiostrongylus cantonensis*: an emergent disease in Brazil. Mem Inst Oswaldo Cruz. (2014) 109:399–407. 10.1590/0074-0276140023 25075779 PMC4155839

[14] Oliveira KL Simões RO Decanine D. First report of *Achatina fulica* (Bowdich, 1822) naturally infected with nematodes from the families Angiostrongylidae and Rhabditidae in the city of Campo Grande, Mato Grosso do Sul, Brasil. J Neotrop Biol. (2021) 18:83–9. 10.5216/rbn.v18i2.839

